# Co-Culture of Osteoblasts and Endothelial Cells on a Microfiber Scaffold to Construct Bone-Like Tissue with Vascular Networks

**DOI:** 10.3390/ma12182869

**Published:** 2019-09-05

**Authors:** Kouki Inomata, Michiyo Honda

**Affiliations:** Department of Applied Chemistry, School of Science and Technology, Meiji University, 1-1-1 Higashimita, Tama-ku, Kawasaki, Kanagawa 214-8571, Japan

**Keywords:** bone tissue engineering, three-dimensional co-culture, osteoblast, endothelial cell, microfiber scaffold, osteogenesis, angiogenesis

## Abstract

Bone is based on an elaborate system of mineralization and vascularization. In hard tissue engineering, diverse biomaterials compatible with osteogenesis and angiogenesis have been developed. In the present study, to examine the processes of osteogenesis and angiogenesis, osteoblast-like MG-63 cells were co-cultured with human umbilical vein endothelial cells (HUVECs) on a microfiber scaffold. The percentage of adherent cells on the scaffold was more than 60% compared to the culture plate, regardless of the cell type and culture conditions. Cell viability under both monoculture and co-culture conditions was constantly sustained. During the culture periods, the cells were spread along the fibers and extended pseudopodium-like structures on the microfibers three-dimensionally. Compared to the monoculture results, the alkaline phosphatase activity of the co-culture increased 3–6 fold, whereas the vascular endothelial cell growth factor secretion significantly decreased. Immunofluorescent staining of CD31 showed that HUVECs were well spread along the fibers and formed microcapillary-structures. These results suggest that the activation of HUVECs by co-culture with MG-63 could enhance osteoblastic differentiation in the microfiber scaffold, which mimics the microenvironment of the extracellular matrix. This approach can be effective for the construction of tissue-engineered bone with vascular networks.

## 1. Introduction

The reconstruction of structural and functional tissues is highly challenging. The ability of excessively damaged organs and tissues to regenerate themselves is low. Since organs and tissues have different structures and functions, treatment should be selected appropriately in consideration of their biochemical properties and anatomical features. Bone has the potential to regenerate. For instance, an iliac crest, which is used for autologous bone grafting to treat bone fractures, could induce vascularization around the implanted area to promote immediate bone formation [1,2]. However, this method has many limitations, so new approaches should be developed.

Tissue engineering to improve or replace damaged tissue has attracted attention. Typical tissue engineering strategies combine three elements (e.g., cells, growth factors, and scaffolds) to construct three-dimensional tissues to enhance the regenerating capability to the original state [3,4,5]. It is essential for the three-dimensional tissue to maintain its structural functions so that blood vessels supply oxygen and nutrients and remove metabolic wastes. These absences cause low cell viability and cell death around the defects [6,7].

Bone tissue is based on a sophisticated system of mineralization and vascularization. In bone, blood vessels are indispensable for the maintenance of bone homeostasis, i.e., the balance between bone formation and bone resorption [8,9,10]. Extensive vascular networks in bone play a significant role in not only providing oxygen and nutrients and draining wastes but also in transporting inorganic ions (e.g., calcium and phosphate ions) necessary for calcification. In the current study, Grüneboom and their colleagues found the existence of an effective communication between the bone marrow vascular system and external circulation. The blood vessels in the periosteum and on the surface of bone connected periosteal circulation, mediated the recruitment of immune cells to the circulation [11]. 

In this context, diverse biomaterials compatible with osteogenesis and angiogenesis have been proposed and developed, such as porous ceramics, hydroxyapatite-based nanocomposites, and bioactive glasses [12,13,14]. However, all materials have not been necessarily designed for the cell culture. For this reason, it is essential to consider cellular characteristics when constructing three-dimensional cell culture scaffolds.

In bone tissue engineering, two interactions are key in the construction of tissue-engineered bone. These interactions are between bone formation cells (osteoblasts) and blood vessel constitution cells (vascular endothelial cells) and between the above-mentioned cells and biomaterials. Previous studies have shown that co-cultures of osteoblastic cells with endothelial cells resulted in the stimulation of osteoblast differentiation and the formation of microcapillary-like structures [15,16]. The adequate adhesion of the cells to the scaffold also has an important role in the expression of normal cell functions, such as cell survival, proliferation, and differentiation [17,18]. Above all, porous scaffolds have attracted attention due to their potential to support vascular tube formation [19,20]. However, those studies have not revealed the interaction between the co-cultured osteoblast, endothelial cells, and the porous scaffolds.

In the present study, osteoblasts were co-cultured with endothelial cells on a microfiber scaffold to examine the characteristics of the co-culture system, such as cell attachment, survival, osteoblast differentiation, and endothelial cell tube formation, which are regarded as important in bone tissue engineering. This model has shown that the three-dimensional co-culture system could be effective in regulating cell survival, adhesion, osteoblastic differentiation, and vascular tube formation. Therefore, the approach would allow for the production of tissue-engineered bone with vascular networks *in vitro*.

## 2. Materials and Methods

### 2.1. Materials

Chemicals and reagents were purchased from the following manufacturers. Eagle’s minimum essential medium (EMEM), non-essential amino acid solution, trypsin-EDTA, monoclonal mouse anti-vinculin antibody (hVIN-1), and CelLytic^TM^ cell lysis reagent were purchased from Sigma-Aldrich (St. Louis, MO, USA). Endothelial cell growth medium 2 kit (ECGM2) was purchased from Takara Bio (Shiga, Japan). Fetal bovine serum (FBS), penicillin, streptomycin, phosphate-buffered saline (PBS, sodium chloride, potassium chloride, disodium hydrogenphosphate, and potassium dihydrogen phosphate), dimethyl sulfoxide (DMSO), 4% paraformaldehyde in PBS, Triton X-100, bovine serum albumin (BSA), 25% glutaraldehyde solution, LabAssay^TM^ ALP, and protein assay Bradford reagent were purchased from FUJIFILM Wako Pure Chemical (Osaka, Japan). Alexa Flour^®^ 488-labeled phalloidin, Alexa Flour^®^ 594-labeled goat anti-mouse IgG_1_, and alamarBlue^®^ cell viability reagent were purchased from Invitrogen (Carlsbad, CA, USA). 3-(4,5-dimethyl-2-thiazolyl)-2,5-diphenyl-tetrazolium bromide (MTT) and 4′,6-diamino-2-phenylindole (DAPI) were purchased from Dojindo (Kumamoto, Japan). Monoclonal mouse anti-human CD31 antibody was purchased from Dako (Glostrup, Denmark). Human VEGF quantikine ELISA kit was purchased from R&D Systems (Minneapolis, MN, USA). MG-63 cells were obtained from ATCC^®^ CRL-1427^TM^ (Manassas, VA, USA). Human umbilical vein endothelial cells (HUVECs) were obtained from PromoCell (Heidelberg, Germany). The microfiber scaffold was kindly supplied by ORTHO ReBIRTH (Kanagawa, Japan).

### 2.2. Fabrication of the Microfiber Mesh Scaffolds

The microfiber mesh scaffolds consisted of 30 wt.% of poly (lactic-co-glycolic acid) (PLGA, LG855S, Evonik Japan, Tokyo, Japan), 40 wt.% of β-tricalcium phosphate (β-TCP, β-TCP-100, Taihei Chemical Industrial Co., LTD, Osaka, Japan), and 30 wt.% of silicon-doped vaterite (SiV) powders [21]. The above composites were dissolved in 8 wt.% of chloroform for electrospinning. Electrospinning was carried out using a nanofiber electrospinning system (NANON-03, MECC Co., LTD. Fukuoka, Japan). PLGA/β-TCP/SiV solution was put into a syringe (diameter: 15.8 mmφ, volume: 10 mL) and pumped out of the syringe at a rate of 4 mL h^−1^. A voltage supplier was used to maintain the voltage at 24 kV. All experiments were carried out at room temperature and samples were also dried at room temperature for overnight. The microfiber mesh scaffolds for the cell culture were sterilized by gamma ray irradiation.

### 2.3. Cell Culture

In this study, human osteoblast-like MG-63 cells and HUVECs were used as models of osteoblasts and endothelial cells. The MG-63 cells were cultured in EMEM supplemented with 10% heat-inactivated FBS, 1% non-essential amino acid solution, 100 U/mL penicillin, and 100 μg/mL streptomycin. The HUVECs were cultured in ECGM2. Both the MG-63 cells and HUVECs were grown under a humidified atmosphere containing 5% CO_2_ at 37 °C. All experiments were conducted using the MG-63 cells at passage 3–6 and the HUVECs at passage 1–3.

### 2.4. Co-Culture of the MG-63 Cells and HUVECs on the Microfiber Scaffold

The MG-63 cells and HUVECs were suspended in a ratio of 1:4 with ECGM2, according to a previously described method [14]. Before cell seeding, a microfiber scaffold was placed in each well of a 24-well plate and hydrophilized with growth medium for 30 min in a humidified atmosphere containing 5% CO_2_ at 37 °C. The suspended cells (5.0 × 10^4^ or 5.0 × 10^5^ cells) were seeded on each scaffold. As a control, MG-63 cells were seeded alone in EMEM at the same density. The culture medium was refreshed every two days, and the culture was maintained for a maximum of 14 days.

### 2.5. Initial Cell Attachment

Initial cell attachment was determined using an MTT-based assay, according to the manufacturer’s protocol. At 6 h after incubation, the MTT reagent was added to the medium (final concentration of 0.5 mg/mL) and incubated for 4 h. The stain was then eluted with DMSO and centrifuged. The absorbance was measured at 570 nm (measurement wavelength) and 650 nm (reference wavelength) using a microplate reader (Multiskan FC, Thermo Fisher Scientific, Waltham, MA, USA). Initial cell attachment was calculated by the ratio between the absorbance of the cells that adhered to the scaffolds and the absorbance of the cells cultured without the scaffold.

### 2.6. Cell Viability

Metabolic activity of the cells on the scaffolds was assessed as cell viability using a resazurin-based assay, according to the manufacturer’s procedure. At 5, 7, and 14 days, the medium was exchanged for growth medium containing 10% alamarBlue^®^ reagent and incubated for 4 h. The absorbance was measured at 570 nm (measurement wavelength) and 595 nm (reference wavelength) using a microplate reader. The percent of reduced alamarBlue^®^ reagent was calculated as previously described [22].

### 2.7. Immunofluorescence Microscopy

The cells were washed twice with PBS and fixed with 4% paraformaldehyde in PBS for 15 min at room temperature. The cells were then permeabilized with 0.1% Triton X-100 in PBS for 15 min at room temperature. After rinsing with PBS twice, the cells were blocked with 3% BSA in PBS for 1 h at room temperature and incubated with a primary antibody, monoclonal mouse anti-vinculin antibody (1:400), or monoclonal mouse anti-human CD31 antibody (1:250), which was diluted in PBS containing 3% BSA overnight at 4 °C. The cells were washed with PBS twice and then stained with Alexa Flour^®^ 488-labeled Phalloidin (1:250) for F-actin, Alexa Flour^®^ 594-labeled goat anti-mouse IgG_1_ (1:500) for vinculin, or CD31 and DAPI (1:500) for the nucleus diluted in PBS for 1 h at room temperature in the dark. The cells were washed with PBS and then examined by florescence microscopy (BZ X-710, Keyence, Osaka, Japan).

### 2.8. Scanning Electron Microscopy

The cells were washed twice with PBS and fixed with 2.5% glutaraldehyde in PBS overnight at 4 °C. After rinsing with PBS twice, the cells were freeze-dried for three days. The specimens were coated with gold using sputtering and then examined by scanning electron microscopy (SEM, VE-9800, Keyence, Osaka, Japan) at an accelerating voltage of 5 kV.

### 2.9. Measurements of Alkaline Phosphatase and Vascular Endothelial Growth Factor

Alkaline phosphatase (ALP) activity and vascular endothelial growth factor (VEGF) secretion were measured similarly to the method described in previous studies [15,23]. At 5, 7, and 14 days, the scaffolds and supernatants were collected and stored at −80 °C. The cells were lysed in CelLytic^TM^ M and homogenized by sonication. The cell lysates were centrifuged and assayed for ALP activity using a colorimetric analysis using *p*-nitrophenyl phosphate as the substrate following the protocol of LabAssay^TM^ ALP. One unit was defined as the activity that produced one nanomole of *p*-nitrophenol after 15 min. The supernatants were assessed for VEGF secretion using the sandwich enzyme-linked immunosorbent assay (ELISA), according to each manufacturer’s instructions. Total protein concentrations were determined by the Bradford standard method.

### 2.10. Statistical Analysis

The data were statistically analyzed for determination of the mean and the standard deviation (SD) of the mean. The Student’s *t*-test was carried out with a significance level of *p* < 0.05.

## 3. Results

### 3.1. Initial Cell Attachment and Cell Morphology

**Figure 1:**
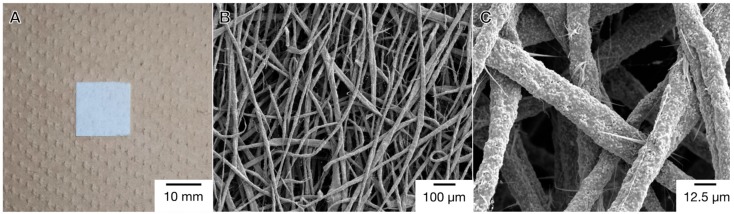
Light photomicrograph (**A**) and SEM images (**B**,**C**) of a microfiber mesh scaffold. Bars indicate 10 mm (**A**), 100 µm (**B**), and 12.5 µm (**C**).

In the present study, we used a microfiber scaffold, which was composed of a three-dimensional porous matrix (Figure 1). The scaffold consisted of random fibers with an average fiber diameter of 10 to 30 μm. The three-dimensional microfiber structure in the scaffold might be a suitable geometry for cell growth and formation of vascular networks.

**Figure 2:**
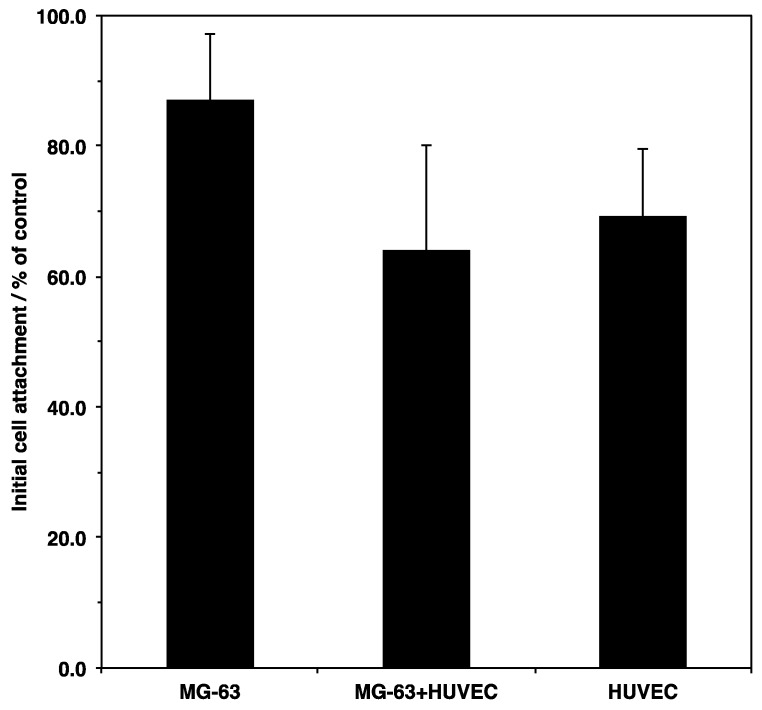
Initial cell attachment of MG-63 cells and/or HUVECs on a microfiber mesh scaffold. Cells were seeded at 5.0 × 10^5^ cells cm^−3^ on the scaffold placed in each well of a 24-well plate and cultured for 6 h. Initial cell attachment was assessed using an MTT assay described in Materials and Methods and calculated by the ratio between the absorbance of the cells that adhered to the scaffold and the absorbance of the cells cultured without the scaffold. Data were determined from three replicate samples and are shown as mean ± SD. There were no significant differences among them.

Initial cell attachment is a key index for evaluating the biocompatibility of materials. The interaction between cells and biomaterials contributes to cell activity on the scaffold, such as cell survival, proliferation, and differentiation. To examine the initial attachment on the porous microfibers, the cells were seeded on the scaffold and incubated for 6 h. The percentage of adherent cells was then determined using an MTT assay (Figure 2). The results showed that more than 60% of the cells adhered to the scaffold were compared to the culture plate, regardless of the cell type and culture conditions. Osteoblasts showed the highest attachment among all the cell types. However, there were no significant differences depending on cellular types and culture conditions. The geometry and surface properties of the scaffold might induce higher initial attachment.

**Figure 3:**
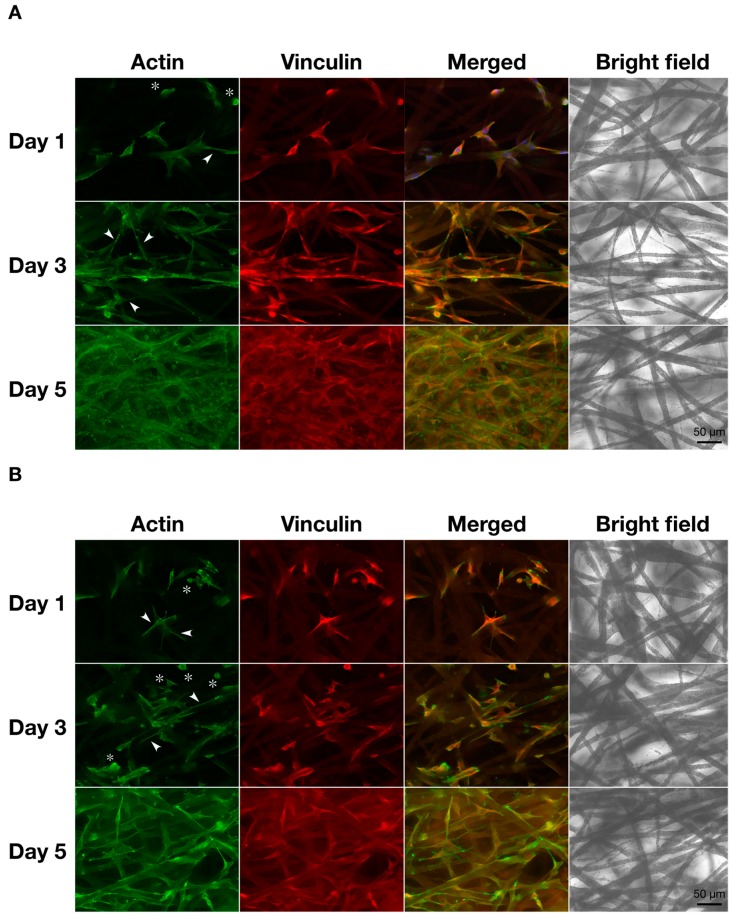
Morphological observation of (**A**) monoculture and (**B**) co-culture cells by immunofluorescence microscopy. Cells were seeded at 5.0 × 10^4^ cells cm^−3^ on a microfiber mesh scaffold placed in each well of a 24-well plate and cultured for 1, 3, and 5 days. At the culture time, the cells were fixed and stained with Alexa Flour^®^ 488-labeled phalloidin for actin (green) and anti-vinculin for vinculin (red). They were viewed through a fluorescence phase-contract microscope at 20× magnifications (scale bars: 50 µm). Arrowheads show the spindle-shaped cells and asterisks indicate shrinkage-rounded cells.

**Figure 4:**
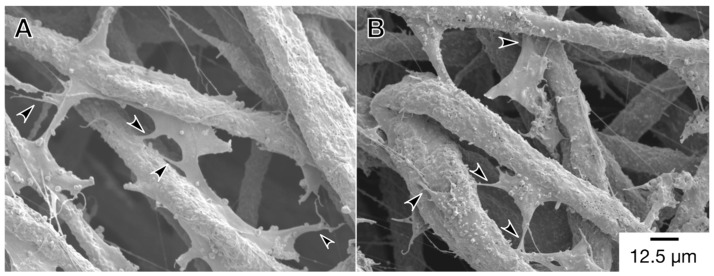
Morphological observation of (**A**) monoculture and (**B**) co-culture cells by SEM. Cells were seeded at 5.0 × 10^4^ cells cm^−3^ on a microfiber mesh scaffold placed in each well of a 24-well plate and cultured for five days. At the culture time, the cells were fixed and viewed with SEM at 800× magnifications (scale bar: 12.5 µm). Arrowheads show pseudopodium-like structures.

Next, to investigate the cell adhesion and cell spreading on the scaffold, fluorescence microscopy was used to observe the actin cytoskeleton and vinculin of the cells (Figure 3). These cell behaviors also affect cell activity on the scaffold. Vinculin is an adaptor protein connecting actin filaments with integrin and is then expressed in spreading cells adherent to the extracellular matrix via integrin. Therefore, the immunofluorescent staining of vinculin can be used to evaluate cell adhesion on the scaffold. On the first day after seeding, the cells had exhibited both spindle-shaped and shrinkage-rounded morphology (Arrowheads and asterisks in Figure 3A,B). As the culture time passed, the cells were well spread, and they expressed vinculin. In addition, to confirm the micro-surrounding of the cells on the scaffold, the cells were observed by SEM (Figure 4). Under each culture condition, we observed two types of cells that were elongating along the fibers and extending pseudopodium-like structures between the fibers (Arrowheads in Figure 4A,B). These results suggested that cells could adhere to the scaffold via integrin. This was supported by previous reports [21,24,25].

### 3.2. Cell Viability

**Figure 5:**
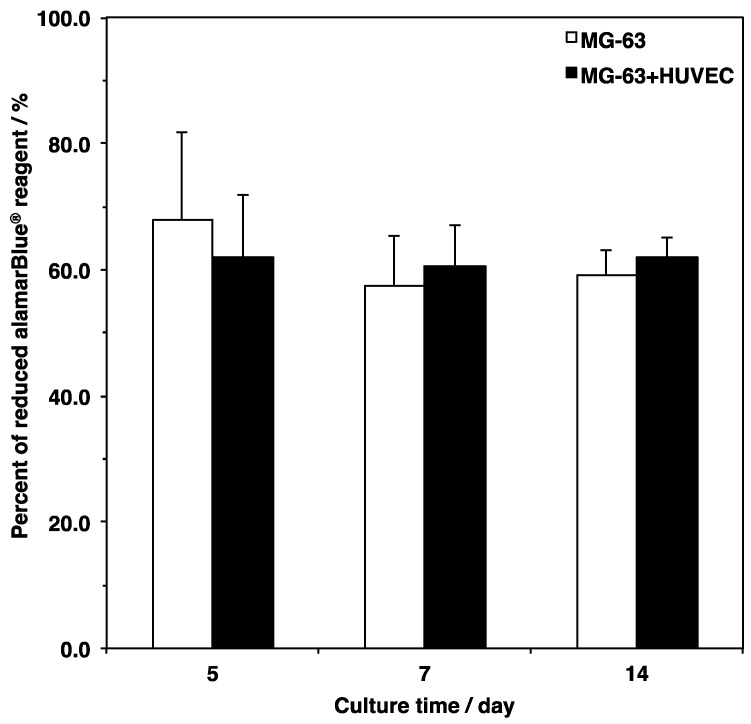
Cell viability of monoculture and co-culture on a microfiber mesh scaffold. Cells were seeded at 5.0 × 10^5^ cells cm^−3^ on the scaffold placed in each well of a 24-well plate and cultured for 5, 7, and 14 days. Cell viability was assessed using an alamarBlue^®^ assay described in Materials and Methods. Data were determined from three replicate samples and are shown as mean ± SD. There were no significant differences among them.

Three-dimensional cell culture scaffolds are designed for the construction of engineered tissue with biomimetic environments ex vivo. Cell survival on biomaterials is important for supposing cell behavior under similar situations in vivo. In the present study, the metabolic activity of the cells cultured on the scaffold was measured using a resazurin-based assay (Figure 5). Cell viability in the monoculture of the MG-63 cells slightly decreased from day 5 to day 7, even though there was no significant difference between the culture periods. On the other hand, the viability in co-culture of MG-63 cells HUVECs was constantly sustained. Comparing the monoculture and co-culture cells, no significant differences between culture conditions were observed. These results showed that the porous and fibrous scaffold could support cell survival by serving as a pathway for nutrients and oxygen [26,27].

### 3.3. Osteogenic Differentiation

**Figure 6:**
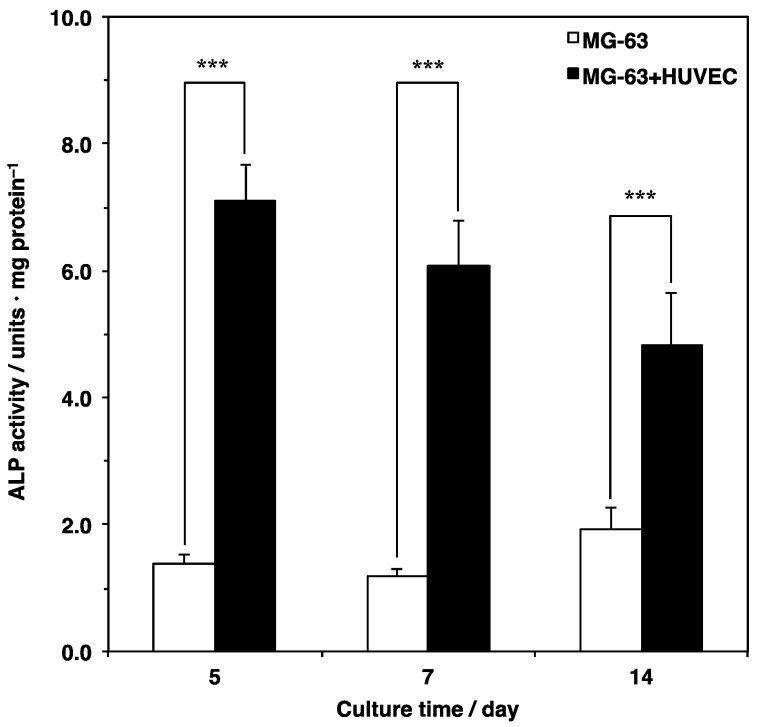
Comparison of ALP activity in a monoculture and a co-culture on a microfiber mesh scaffold. Cells were seeded at 5.0 × 10^5^ cells cm^−3^ on the scaffold placed in each well of a 24-well plate and cultured for 5, 7, and 14 days. ALP activity was assessed using a *p*-nitrophenyl phosphate substrate assay described in Materials and Methods. Data were determined from three replicate samples, which are shown as mean ± SD. *** *p* < 0.001 compared with the monoculture (MG-63 cells alone).

Previous studies have shown that co-culturing osteoblasts with endothelial cells enhances osteogenesis [15,16]. To investigate the effects on osteoblast differentiation by co-culture of osteoblasts and endothelial cells on the scaffold, bone-specific protein alkaline phosphatase (ALP) activity was quantitated using a *p*-nitrophenyl phosphate substrate assay (Figure 6). The ALP activity in the MG-63 monoculture was constantly sustained from day 5 to day 7 and slightly increased from day 7 to day 14. However, there were no significant differences between the culture times. In contrast, the ALP activity in the co-culture of the MG-63 cells and HUVECs was remarkably high during all culture periods. Comparing the two, the ALP activity under the co-culture conditions was three to six times higher than that under the monoculture conditions. These results indicated that endothelial cells could also enhance osteoblast differentiation on the fibrous scaffold.

### 3.4. Angiogenic Properties

**Figure 7:**
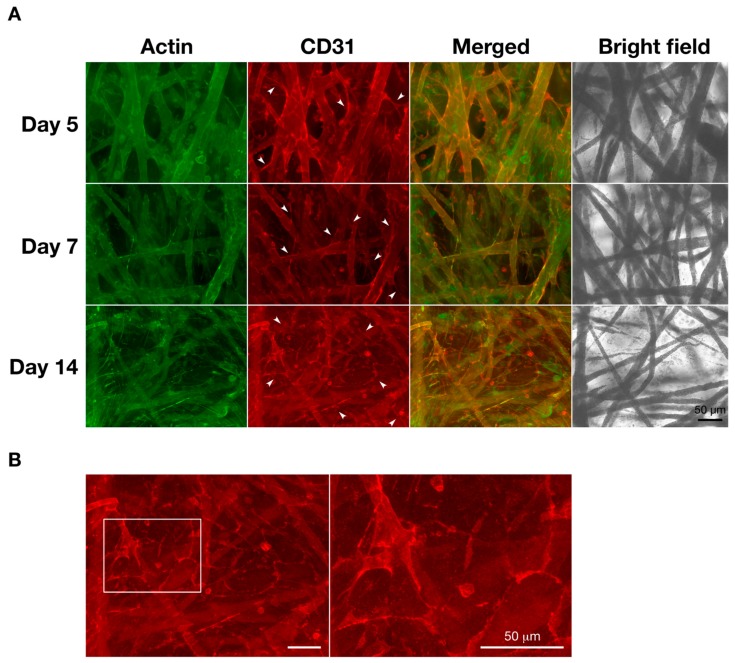
Morphological observation of monoculture and co-culture cells by immunofluorescence microscopy. Cells were seeded at 5.0 × 10^5^ cells cm^−3^ on a microfiber mesh scaffold placed in each well of a 24-well plate and cultured for five, seven, and 14 days. At the culture time, the cells were fixed and stained with Alexa Flour^®^ 488-labeled phalloidin for actin (green) and anti-human CD31 for CD31 (red). They were viewed through a fluorescence phase-contract microscope at 20× magnifications (scale bars: 50 µm). Arrowheads (**A**) show microcapillary-like structures and square enclosure (**B**) indicates a vascular network area.

In bone tissue engineering, the lack of functional microvasculature induces the deficient supply of oxygen and nutrients and decreases the removal of metabolic wastes. This leads to low cell viability and cell apoptosis, which results in clinical problems [7,28,29]. To examine the vascularization in the co-culture system on the microfiber scaffold, the actin cytoskeleton and CD31 of the cells were observed by fluorescence microscopy (Figure 7). CD31 is well known as the platelet endothelial cell adhesion molecule-1 (PECAM-1) and a specific marker of endothelial cells that can discriminate between MG-63 cells (CD31-negative cells) and HUVECs (CD31-positive cells). On the other hand, F-actin, which is a cytoskeleton protein, is expressed in both osteoblasts and endothelial cells. Five days after seeding, the HUVECs were distributed along the fibers. After seven days, the HUVECs were well spread between the fibers and formed microcapillary-like structures (Arrowheads in Figure 7A). At 14 days of culture, the cells filled in the spaces where the fibers were intertwined and vascular networks were formed among the HUVECs (Figure 7B).

**Figure 8:**
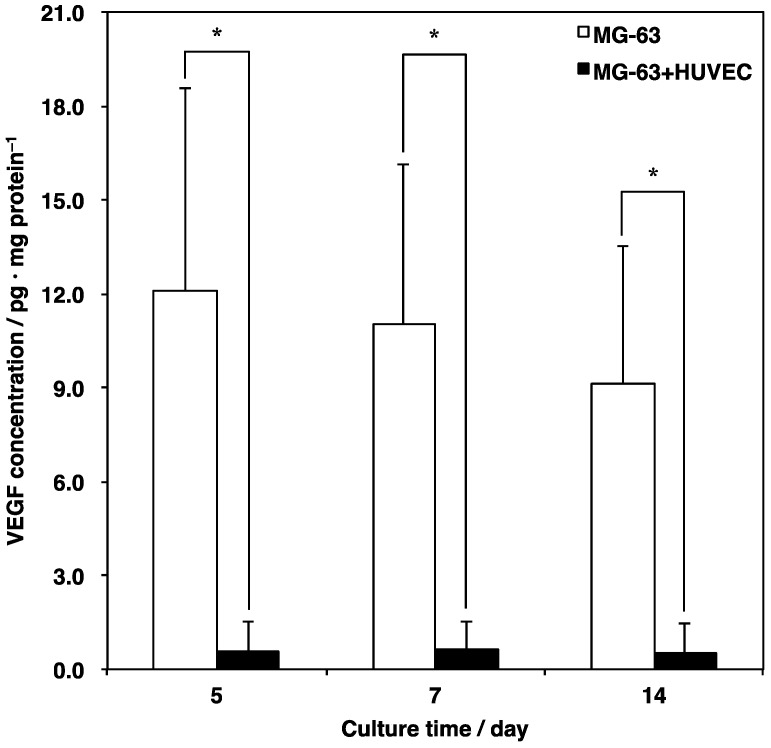
Comparison of vascular endothelial growth factor (VEGF) secretion in a monoculture and a co-culture on a microfiber scaffold. Cells were seeded at 5.0 × 10^5^ cells cm^−3^ on the scaffold placed in each well of a 24-well plate and cultured for five, seven, and 14 days. VEGF production was assessed using a sandwich ELISA described in Materials and Methods. Data were determined from three replicate samples, which are shown as mean ± SD. * *p* < 0.05 compared with monoculture (MG-63 cells alone).

Next, to confirm the process of angiogenesis in detail, VEGF, which is a growth factor that positively contributes to vascularization, was quantified using a sandwich ELISA (Figure 8). In the MG-63 monoculture, high VEGF secretion was detected at all culture periods. On the other hand, the concentration of VEGF in the co-culture of the MG-63 cells and HUVECs significantly decreased compared with the monoculture. These results suggested that the HUVECs might consume the VEGF, which is mainly produced by MG-63 cells to form the vascular lumens.

## 4. Discussion

The effective introduction of blood vessels into engineered tissue is a common challenge in tissue engineering. To date, diverse biomaterials have been developed in consideration of cellular distribution and penetration [20,30]. When pores are inappropriately filled with excess cells, decreased cell survival and necrosis can be induced. However, co-culturing target cells with endothelial cells could be employed to avoid such situations. Additionally, endothelial cells can form vascular networks to supply oxygen and nutrients and remove metabolic wastes in engineered tissue. In bone tissue engineering, a co-culture system of osteoblasts with endothelial cells on porous scaffolds has already been proposed [8,26]. However, the interaction between the co-culture cells and the scaffold was unclear.

In the present study, the processes of osteogenesis and angiogenesis in a co-culture of osteoblasts and endothelial cells on a microfiber scaffold was investigated, and cell attachment, extension, survival, osteoblastic differentiation, and endothelial cell tube formation were the measured variables. A porous and fibrous scaffold was adopted. The scaffolds were prepared using PLGA, β-TCP, and SiV composites, and they had *in vivo* biological properties such as biocompatibility, bio-resorbability, and osteo-conduction [21,24,31]. In particular, PLGA has a property that complements hydrophilicity and hydrophobicity to support cell attachment. The fibrous structure composed of PLGA contributes to cell survival by serving as the pathways for nutrients and oxygen [20,26,27]. Our results showed that the percentage of adherent cells was more than 60%, and their cell viabilities were constantly sustained regardless of the cell types and culture conditions. It is probable that these cell activities were supported by the geometry and surface properties of the scaffold. As previously reported, electro-spun scaffolds could provide the three-dimensional interconnectivities that allow integration between cells and fibers [32,33]. In random fiber networks with large pores, cells could easily integrate inside the scaffold. In this study, a microfiber scaffold with random networks could provide an appropriate environment for cell proliferation and the circularity of oxygen and nutrients. Additionally, a large surface area, which has higher quantity of protein adsorption, could enhance the cell adhesion [34]. 

Bone-specific ALP is a marker of osteoblast differentiation *in vitro*, which is known to be expressed until maturation. The ALP activity in the co-culture of the MG-63 cells and HUVECs was approximately three to six times higher than that in the monoculture of the MG-63 cells. These results were consistent with previous studies [15,16,35]. In addition, some studies have suggested that the up-regulation of ALP activity could differ between direct and indirect cell cultures [36,37,38]. This would mean that endothelial cells support osteoblasts via different pathways in proximal and remote conditions. Since the ALP activity on the scaffold was between the effects of direct and indirect contact in the two-dimensional culture, the co-culture on the fibrous scaffold can potentially reflect proximal and remote conditions. However, the mechanisms lack detail.

Previous studies demonstrated that co-culturing osteoblasts and endothelial cells enhances angiogenesis as well as osteogenesis [15,16]. In the present study, we also observed that vascular lumens were formed among the HUVECs over time. Some studies have suggested that osteoblasts could be a main source of VEGF in co-culture systems of osteoblasts and endothelial cells [36,39,40]. In fact, the concentration of VEGF under the co-culture condition significantly decreased when compared with the MG-63 cell monoculture. These results indicate that HUVECs might consume MG-63 cell-derived VEGF to drive cell activity such as proliferation, survival, migration, and tube formation.

The co-culture of osteoblasts and endothelial cells on a scaffold is a dynamic system based on two interactions, which are between osteoblasts and endothelial cells as well as between these cells and the scaffold. Osteoblasts and endothelial cells support and enhance each other’s cell behavior via three main elements (e.g., humoral factors, cell junctions, and extracellular matrixes) [41,42,43,44]. The importance of cell communication is divided between direct and indirect culture. For instance, direct contact between osteoblasts and endothelial cells can be a starting point for gap junction formation to up-regulate osteogenesis and angiogenesis. However, plostanoids negatively modulates the VEGF-mediated crosstalk between osteoblasts and endothelial cells in direct contact [36,37]. The co-culture of the MG-63 cells and HUVECs on the microfiber scaffold can reflect both remote effects (via humoral factors) and proximal effects (via cell junctions) from the perspective of ALP activity, vascular lumen formation, and VEGF secretion. Therefore, these results suggest that the VEGF produced mainly by the MG-63 cells could be consumed by binding to the VEGF receptor on the HUVECs to contribute to signaling pathways. As a result, the activation of HUVECs can contribute to the behavior of the MG-63 cells, which results in the enhancement of osteoblastic differentiation. However, the pathways have not yet been identified.

It has been reported that cell adhesion to biomaterials could be involved in osteoblast proliferation and differentiation as well as endothelial cell tube formation [45,46]. The cells in this study exhibited spindle-shaped and shrinkage-rounded morphologies at 1 day after seeding, even though the cells exhibited the spread form and pseudopodium-like structures over time. Furthermore, in the case of large cell numbers, the HUVECs distributed themselves along and between the fibers, which resulted in the formation of microcapillary-like structures in three dimensions. On the other hand, it is known that insufficient cell attachment and penetration cannot effectively induce osteogenesis and angiogenesis in the case of dense ceramics and metallics [46]. Therefore, it is considered that the fibrous scaffolds can regulate the vascularization topologically. However, additional studies are warranted to elucidate the interactions between osteoblasts and endothelial cells and between these cells and scaffolds. Furthermore, histological analyses of the bone repair process using a microfiber scaffold in an animal model would make it possible to provide new findings of osteogenesis and angiogenesis. In particular, the histomorphometric analyses of bone structure and remodeling (e.g., bone volume, trabecular thickness, bone mineral density, and bone formation rate) would indicate valuable information about bone metabolism [47].

In summary, the co-culture of osteoblasts with endothelial cells on a microfiber scaffold stimulated cell activity including cell adhesion, survival, osteoblastic differentiation, and endothelial cell tube formation via cell–cell communication and cell–scaffold interaction. Therefore, this approach highlights tissue-engineered bone with vascular networks by crosstalk between osteoblasts and endothelial cells and the interaction between these cells and the scaffold.

## 5. Conclusions

The reconstruction of three-dimensional tissue requires consideration of the biochemical properties and anatomical features of objective tissue. In tissue engineering, it is essential to investigate the biological properties of the scaffold such as cell attachment, adhesion, extension, survival, proliferation, and differentiation. The induction of vascularization into engineered tissue is also key to avoid clinical issues caused by implanted tissue. In the present study, osteoblasts were co-cultured with endothelial cells on a microfiber biomaterial to examine the processes of osteogenesis and angiogenesis on the scaffold. Our results showed that the cells could attach and spread with the formation of pseudopodium-like structures and that the cells constantly sustained their own viability. Furthermore, osteoblasts and endothelial cells could enhance and improve each other’s functions such as osteoblastic differentiation and endothelial cell tube formation. These results were supported by the properties of the scaffold such as fibrous and topological structures. However, (i) cell-to-cell signal transduction on the microfiber scaffold and (ii) the potential of bone repair after implantation of the scaffold are still unclear. Further studies will provide new insights into angiogenesis in bone remodeling and bone metabolism. Taken together, this model has shown that the three-dimensional co-culture system could regulate both osteogenesis and angiogenesis to effectively construct bone-like tissue with vascular networks in vitro. 

## Abbreviations

EMEM	Eagle’s minimum essential medium
ECGM2	endothelial cell growth medium 2 kit
FBS	fetal bovine serum
PBS	phosphate-buffered saline
DMSO	dimethyl sulfoxide
BSA	bovine serum albumin
MTT	3-(4,5-dimethyl-2-thiazolyl)-2,5-diphenyl-tetrazolium bromide
DAPI	4′,6-diamino-2-phenylindole
HUVEC	human umbilical vein endothelial cell
SEM	scanning electron microscopy
ALP	alkaline phosphatase
VEGF	vascular endothelial growth factor
ELISA	enzyme-linked immunosorbent assay
PECAM-1	platelet endothelial cell adhesion molecule-1
β-TCP	β-tricalcium phosphate
PLGA	poly (lactic-co-glycolic acid)
SiV	siloxane-doped vaterite
